# Preoperative Systemic Inflammatory Indices (SII, SIRI and PIV) Associated with Adverse Histology and 12-Month Recurrence After Transurethral Resection of Non-Muscle-Invasive Bladder Cancer

**DOI:** 10.3390/jcm15218536

**Published:** 2026-11-02

**Authors:** Dragoș Florin Vasile, Mihnea Meșină, George G. Mitroi, Alexandru-Daniel Danilă, Cosmin Vasile Obleaga, Mihai Alexandru Radu, Georgică Târtea, George F. Mitroi

**Affiliations:** 1 Department of Urology, University of Medicine and Pharmacy of Craiova, 2 Petru Rareș Street, 200349 Craiova, Romania; dragos.vasile@umfcv.ro (D.F.V.); mihneamesina@yahoo.com (M.M.); 28radu.mihai@gmail.com (M.A.R.); george.mitroi@umfcv.ro (G.F.M.); 2 Department of Dermatology, University of Medicine and Pharmacy of Craiova, 2 Petru Rareș Street, 200349 Craiova, Romania; george.mitroi94@umfcv.ro; 3 Department of Urology, County Emergency Hospital of Târgu-Jiu, 18 Progresului Street, 210218 Târgu-Jiu, Romania; danila@urologtargujiu.ro; 4 Department of Surgical Oncology, University of Medicine and Pharmacy of Craiova, 2 Petru Rareș Street, 200349 Craiova, Romania; 5 Experimental Research Centre for Normal and Pathological Aging, University of Medicine and Pharmacy of Craiova, 2 Petru Rareș Street, 200349 Craiova, Romania; georgica.tartea@umfcv.ro; 6 Department of Cardiology, University of Medicine and Pharmacy of Craiova, 2 Petru Rareș Street, 200349 Craiova, Romania

**Keywords:** non-muscle-invasive bladder cancer, TURBT, systemic inflammation response index, systemic immune–inflammation index, pan-immune–inflammation value, recurrence

## Abstract

**Background/Objectives:** Non-muscle-invasive bladder cancer (NMIBC) recurs frequently, and clinicopathological tools predict recurrence imperfectly. We assessed whether six preoperative blood-count-derived inflammatory indices (SII, SIRI, PIV, NLR, PLR, and LMR) are associated with adverse histology and 12-month recurrence and whether SIRI adds prognostic information to established risk factors. **Methods:** We retrospectively analysed 180 consecutive NMIBC patients treated by transurethral resection of bladder tumour (TURBT) and immediate instillation. Indices were modelled per 1-SD in logistic (adverse histology: high grade, T1 or carcinoma in situ) and Cox (recurrence) models adjusted for established risk factors. **Results:** Adverse histology occurred in 121 patients (67.2%); 88 recurred (12-month Kaplan–Meier estimate: 49.2%). All six indices were univariably associated with both endpoints, with similar effects (SIRI: adverse histology OR: 2.35, 95% CI: 1.41–3.90; recurrence HR: 1.41, 1.19–1.67). After adjustment, SIRI was not independently associated with adverse histology (OR: 1.69, 0.92–3.11) or recurrence (HR: 1.10, 0.86–1.40) and did not improve discrimination (ΔC: 0.001, −0.006 to 0.016). Results were robust across sensitivity analyses, including propensity weighting for BCG (limited by positivity violations). **Conclusions:** SIRI added no demonstrable independent prognostic value. Given modest overfitting (optimism-corrected calibration slope: 0.79), neither the indices nor the model should be used clinically without external validation and recalibration.

## 1. Introduction

Bladder cancer is one of the most frequently diagnosed malignancies of the urinary tract [1], and roughly three quarters of new cases present as non-muscle-invasive bladder cancer (NMIBC) [2]. Transurethral resection of the bladder tumour (TURBT) removes the visible lesion, but NMIBC is defined clinically by its strong tendency to recur and, in a proportion of patients, to progress to muscle-invasive disease [3]. This behaviour commits patients to prolonged cystoscopic surveillance and, frequently, to adjuvant intravesical therapy, generating a substantial clinical and economic burden.

Risk stratification currently relies on clinicopathological variables, including tumour grade, stage, size, multifocality, prior recurrence rate and concomitant carcinoma in situ (CIS). The European Organisation for Research and Treatment of Cancer (EORTC) scoring models are used to estimate recurrence risk in defined NMIBC populations, whereas contemporary European Association of Urology (EAU) risk groups are primarily used to stratify progression risk [2,4]. These tools remain the clinical reference framework but offer imperfect discrimination for the individual patient, leaving room for objective, inexpensive and reproducible biomarkers. The quality and completeness of the initial resection are themselves important determinants of recurrence [5], and we have previously shown that intraoperative control of the obturator reflex is associated with specimen quality and early recurrence after TURBT [6]. Beyond such technical determinants, host-related factors—particularly the systemic inflammatory response—may also shape tumour behaviour and prognosis.

Chronic inflammation is an established hallmark of carcinogenesis [7,8], contributing to tumour initiation, progression and treatment resistance across malignancies [9,10], and the systemic inflammatory state can be quantified from a routine complete blood count. The neutrophil-to-lymphocyte ratio (NLR), platelet-to-lymphocyte ratio (PLR) and lymphocyte-to-monocyte ratio (LMR) were the first such indices to be examined, and an elevated NLR in particular has been associated with adverse outcomes across a range of solid tumours [11,12]. More recently, higher-order composites—the systemic immune–inflammation index (SII), the systemic inflammation response index (SIRI) and the pan-immune–inflammation value (PIV)—have been proposed as more complete descriptors of the balance between pro-tumour inflammation and antitumour immunity, at no additional laboratory cost [13,14,15].

Evidence in NMIBC remains heterogeneous, with variable cut-offs, differing endpoints and inconsistent adjustment for established risk factors [16,17,18]. This study evaluates preoperative SII, SIRI and PIV—alongside NLR, PLR and LMR—as continuous markers associated with adverse histology and 12-month recurrence in a single-centre NMIBC cohort treated by TURBT under a uniform immediate-instillation protocol. In contrast to prior NMIBC series that dichotomised these indices at data-derived thresholds, we model them continuously and assess whether a representative composite index, SIRI, provides incremental prognostic information beyond conventional clinical risk stratification.

## 2. Materials and Methods

### 2.1. Study Design and Population

**Figure 1:**
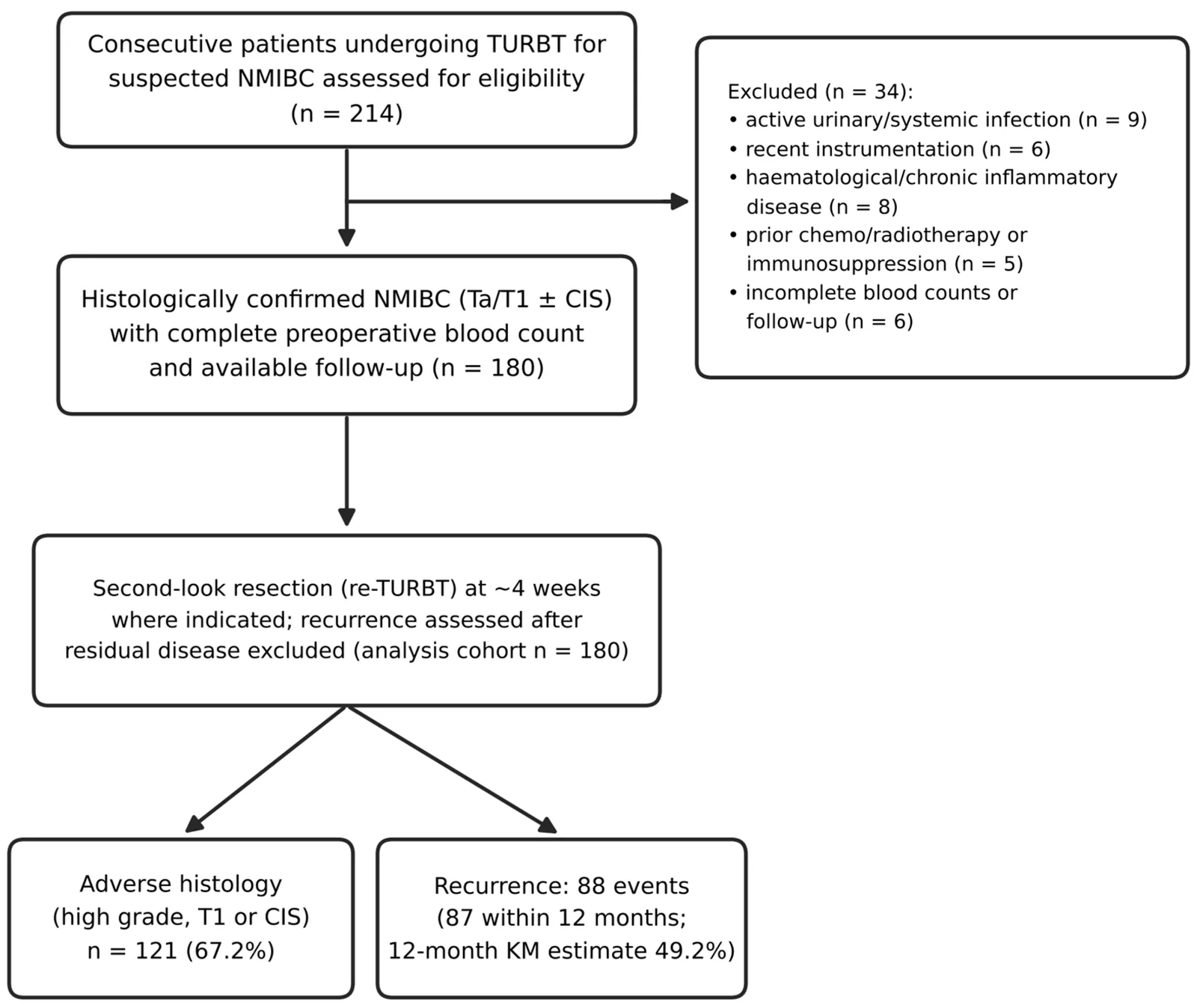
Study flow diagram. Of 214 consecutive patients assessed for eligibility, 34 were excluded (active urinary or systemic infection, n = 9; recent instrumentation, n = 6; haematological or chronic inflammatory disease, n = 8; prior chemotherapy, radiotherapy or immunosuppression, n = 5; incomplete blood counts or follow-up, n = 6), leaving 180 patients for analysis.

In this retrospective observational study, we analysed 180 consecutive patients with histologically confirmed NMIBC who underwent TURBT in the Department of Urology of the Clinical County Emergency Hospital of Craiova between October 2022 and December 2024. Eligible patients had urothelial carcinoma staged Ta or T1, with or without concomitant CIS, complete preoperative haematological data, and available postoperative follow-up data. Patients with active urinary or systemic infection, recent endourological instrumentation before blood sampling, haematological or chronic inflammatory disease, history of chemotherapy, radiation therapy, or immunosuppression were excluded, as these conditions confound systemic inflammatory indices. None of the included patients had systemic corticosteroids, non-steroidal anti-inflammatory drugs, aspirin or statins among the chronic home medications recorded in the medical records; occasional over-the-counter use cannot be fully excluded (see the Limitations section). Tumours were graded according to the 2004/2016 WHO classification (low-grade/high-grade) and staged as per the TNM system. All specimens were examined by the hospital’s department of pathology; no independent central pathology review was performed. The study flow is summarised in Figure 1.

### 2.2. Surgical Protocol

All resections were performed transurethrally. A second-look resection (re-TURBT) was performed approximately four weeks after the initial resection for stage T1 and/or high-grade tumours and whenever detrusor muscle was absent from the initial specimen, according to institutional protocol and EAU recommendations [2]; recurrence was assessed only after residual disease had been excluded at this planned second resection. Postoperative surveillance comprised cystoscopy every three months during the first year. As per institutional protocol, every patient also received a single immediate postoperative intravesical chemotherapy instillation of epirubicin within two hours of the initial resection. Because this instillation was administered uniformly, it was not expected to confound between-group comparisons. Current EAU guidance recommends a single immediate instillation primarily for low-risk and selected intermediate-risk tumours [2]; in our cohort, it did not replace risk-adapted adjuvant therapy, as detailed below. Decisions on further adjuvant intravesical therapy were made at the oncology service and individualised according to the EAU risk group, patient factors and drug availability. All 60 high-risk patients received a further adjuvant course (bacillus Calmette–Guérin [BCG] in 52, intravesical chemotherapy in 8); out of the 100 intermediate-risk patients, 61 received an adjuvant course (chemotherapy in 47, BCG in 14) and 39 were managed with the immediate instillation alone; the 20 low-risk patients received the immediate instillation alone, except for 8 who also received chemotherapy. Overall, 66 patients (36.7%) received adjuvant BCG, 63 (35.0%) adjuvant chemotherapy, and 51 (28.3%) no further adjuvant course. Adjuvant therapy was entered as a three-level covariate (none, chemotherapy, BCG) in the multivariable recurrence models.

### 2.3. Inflammatory Indices

The last complete blood count before surgery was used. Absolute neutrophil (N), lymphocyte (L), monocyte (M) and platelet (P) counts (×10^9^/L) were recorded and the indices computed as: NLR = N/L; PLR = P/L; LMR = L/M; SII = P × N/L; SIRI = N × M/L; and PIV = N × P × M/L.

### 2.4. Endpoints

Two endpoints were prespecified. The first was a composite adverse-histology endpoint, defined as the presence of any of the following: high tumour grade, stage T1 or concomitant CIS (distinct from EAU risk groups, which incorporate additional clinical variables). The composite was prespecified to capture clinically relevant adverse pathological features, each of which independently prompts intensified management (re-TURBT or adjuvant BCG). For a binary outcome, events per variable are determined by the less frequent outcome category; with seven covariates, this ratio was 8.4 for the composite endpoint (59 patients without adverse histology), 12.0 for high grade, 8.6 for stage T1 and 4.7 for concomitant CIS; except for high grade, all fell below the commonly cited guide of about 10 (the CIS model markedly so), so the adjusted estimates for adverse histology are imprecise. The second was recurrence within 12 months, defined as cystoscopically and histologically confirmed tumour recurrence; residual disease identified at the planned re-TURBT was not classified as recurrence. Patients without recurrence were censored at last follow-up. Time-to-recurrence analyses used all available follow-up (maximum: 13 months), so the single recurrence observed after 12 months was included; censoring follow-up at 12 months did not change the results (Supplementary Table S3). The composite endpoint remained the primary adverse-histology endpoint; each component (high grade, stage T1 and concomitant CIS) was analysed separately as a secondary, exploratory analysis, the stage T1 and CIS analyses being based on fewer events and therefore having lower power.

### 2.5. Statistical Analysis

**Figure 2:**
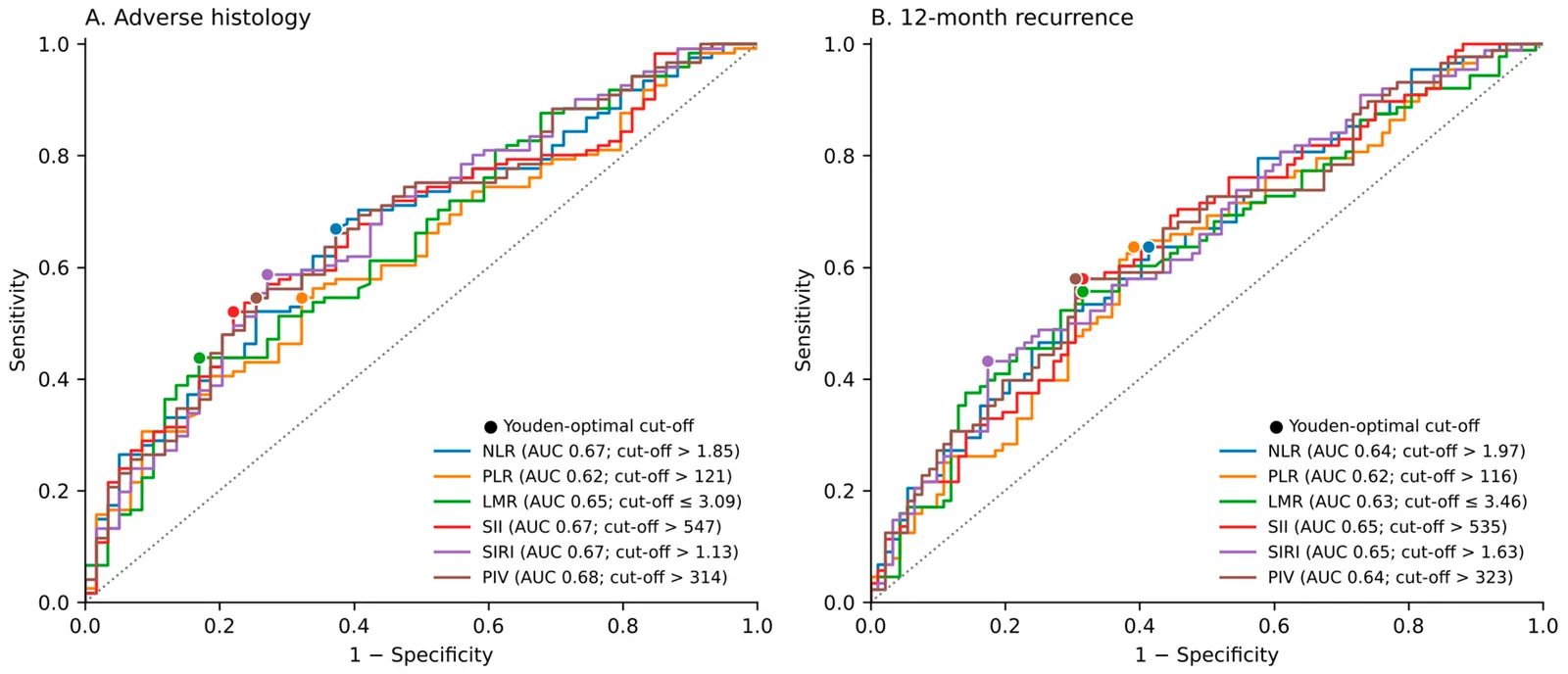
Receiver operating characteristic curves of the six inflammatory indices for (**A**) adverse histology and (**B**) 12-month recurrence; filled circles mark the Youden-optimal descriptive cut-off of each index (Supplementary Table S1); the dotted diagonal line indicates no discrimination (AUC = 0.5).

Continuous variables are summarised as medians (interquartile ranges, IQRs) and compared with the Mann–Whitney U test; categorical variables are expressed as counts (percentages) and compared with the chi-square test (with Yates’ continuity correction for 2 × 2 tables). Discrimination of each index for both endpoints was quantified by the area under the receiver operating characteristic curve (AUC), with the Youden index defining descriptive cut-offs; these internally derived thresholds (marked in Figure 2 and listed in Supplementary Table S1) were not used in any regression model. For association testing, indices were modelled as continuous variables standardised to one standard deviation (per 1-SD), avoiding data-driven dichotomisation; odds ratios (ORs) were obtained by logistic regression for adverse histology and hazard ratios (HRs) by Cox proportional-hazards regression for recurrence. Recurrence-free survival above and below the descriptive cut-offs was displayed by the Kaplan–Meier method; log-rank *p*-values are reported for description only. The incremental value of SIRI over the clinical risk model was assessed by the change in the Harrell C-index [19], with 95% percentile confidence intervals from 2000 bootstrap resamples (for the change in C-index, both models were refitted in each resample), and by a likelihood-ratio test. Because SII, SIRI and PIV are mathematically collinear, they were entered in separate models rather than together. A single composite index was carried forward to the multivariable adverse-histology and recurrence models; SIRI was chosen after the univariable analysis because it had the largest univariable hazard ratio for recurrence among the three composite indices (SII, SIRI and PIV), although SII and PIV had slightly larger univariable odds ratios for adverse histology; the other indices were examined in the full recurrence model as a sensitivity analysis. A clinical risk model was constructed comprising the EAU risk group (low, intermediate or high, assigned in routine practice according to the EAU NMIBC risk-group definitions [2] and recorded in the clinical records, and entered as an ordinal variable, with entry as two indicator variables as a sensitivity analysis; the EORTC recurrence score could not be calculated because the prior recurrence rate required by the EORTC tables [4] was not recorded), adjuvant therapy (as a three-level variable: none, chemotherapy or BCG), tumour size, multifocality, prior recurrence and age; SIRI (per 1-SD) was then added to quantify incremental value. Discrimination was additionally compared by the time-dependent area under the curve at 12 months (cumulative/dynamic definition, with patients event-free at 12 months as controls), estimated with inverse-probability-of-censoring weighting to account for patients censored before 12 months, with a bootstrap confidence interval (refitted models) for its change. Collinearity was assessed by the variance inflation factor, departure from linearity of SIRI by a restricted-cubic-spline/quadratic term, calibration by the bootstrap optimism-corrected calibration slope, and optimism of the C-index by bootstrap. For univariable and multivariable Cox models, the proportional-hazards assumption was assessed with scaled Schoenfeld residuals, with an age-stratified model used as a sensitivity analysis where the assumption was not met, and univariable *p*-values for the six indices were corrected with the Benjamini–Hochberg false-discovery-rate procedure. Among the 180 included patients, there were no missing values in any analysis variable, so imputation was not required. Six otherwise eligible patients (3.2%, 6/186) had been excluded because of incomplete blood counts or follow-up data, so the analysis is a complete-case analysis of eligible patients. Nineteen patients without recurrence had less than 12 months of follow-up; they were censored at last contact rather than treated as missing, which the Cox model accommodates. Sensitivity analyses comprised (i) inverse-probability-of-treatment weighting (IPTW), with the propensity for adjuvant BCG estimated by logistic regression on EAU risk group, tumour size, multifocality, prior recurrence, age and SIRI (weights truncated at the 99th percentile; robust variance); (ii) re-fitting the clinical model with the EAU risk group entered as a binary variable (high vs. low/intermediate risk); (iii) restriction to homogeneously treated subgroups (adjuvant-treated, BCG-treated and high-risk patients); (iv) a granular clinicopathological model in which the EAU risk group and multifocality were replaced by age, sex, tumour size, number of tumours, primary versus recurrent presentation, grade, stage, concomitant CIS and adjuvant therapy; (v) log-transformation of SIRI; (vi) additional adjustment for diabetes, body mass index and current smoking, and replacement of multifocality by the number of tumours; (vii) logistic regression for recurrence by 12 months among the 161 patients with known 12-month status; (viii) truncation of follow-up at 12 months, so that the single recurrence observed after 12 months was censored; (ix) omission of the EAU risk group and, conversely, a model containing only the EAU risk group and tumour size, to assess redundancy between the category and its components (tumour size, multifocality and prior recurrence), together with bootstrap assessment (1000 resamples) of the stability of the SIRI estimate; (x) entry of the EAU risk group as two indicator variables instead of an ordinal term; and (xi) replacement of SIRI by each of the other five indices in the full model. Cox regression was retained as the primary analysis because it uses all 180 patients, accommodates censoring of the 19 patients with less than 12 months of event-free follow-up and incorporates time to recurrence. Apparent calibration at 12 months was displayed graphically by comparing mean predicted probabilities with observed Kaplan–Meier estimates across quartiles of predicted risk; because apparent calibration is optimistic, the calibration slope was corrected for optimism by bootstrap (300 resamples). Post hoc power for the SIRI effect (per 1-SD) in the multivariable Cox model was estimated with Schoenfeld’s method, accounting for its collinearity with the other covariates. A two-sided *p* < 0.05 was considered significant. The study is reported in accordance with the STROBE statement for observational studies [20] (Figure 1). Analyses used Python 3.13 (pandas 2.3, scikit-learn 1.9, lifelines 0.30 and statsmodels 0.15).

## 3. Results

### 3.1. Cohort Characteristics

Of 214 consecutive patients assessed for eligibility, 34 were excluded because of active urinary or systemic infection (n = 9), recent instrumentation (n = 6), haematological or chronic inflammatory disease (n = 8), prior chemotherapy, radiotherapy or immunosuppression (n = 5), or incomplete blood counts or follow-up (n = 6), leaving 180 patients for analysis (Figure 1). The 180 patients had a median age of 68 years (IQR: 62–74), and 136 (75.6%) were men. High-grade tumours were present in 84 (46.7%), stage T1 in 60 (33.3%) and concomitant CIS in 33 (18.3%); 76 tumours (42.2%) were multifocal, and 60 patients (33.3%) had recurrent disease at presentation. By the composite definition, 121 patients (67.2%) had adverse histology, and detrusor muscle was present in 159 specimens (88.3%). All patients received an immediate postoperative instillation. The Kaplan–Meier estimate of recurrence at 12 months was 49.2% (95% CI: 42.1–56.9; 87 events within 12 months); the median follow-up was 11 months (IQR: 6.5–12). A 12-month recurrence status was ascertainable in 161 patients (89.4%; recurrence within 12 months or event-free follow-up of at least 12 months); 19 (10.6%) were censored earlier without recurrence, and one further recurrence occurred at 13 months (88 recurrences in total). Baseline characteristics by recurrence status are shown in Table 1.

### 3.2. Inflammatory Indices and Recurrence

All six indices differed significantly between patients with and without recurrence (Table 2): NLR, PLR, SII, SIRI and PIV were higher, and LMR lower, among those who recurred. The median SIRI was 1.37 (IQR: 0.84–2.22) in recurring patients versus 0.99 (0.56–1.47) in non-recurring patients (*p* < 0.001).

### 3.3. Discrimination and ROC Analysis

On ROC analysis, discrimination was moderate and broadly similar across indices for both endpoints (Figure 2; Supplementary Table S1). For adverse histology, AUCs ranged from 0.62 (PLR) to 0.68 (PIV); for 12-month recurrence, they ranged from 0.62 to 0.65. Recurrence ROC analyses compared the 88 patients with recurrence with the 92 without; restricted to the 161 patients with an ascertainable 12-month status, the AUCs were similar (0.62–0.65) and the SIRI cut-off was unchanged. These AUC differences were modest and should not be interpreted as evidence of statistically superior discrimination without formal pairwise comparison. Sensitivity at the descriptive SIRI recurrence cut-off was limited (0.43; Supplementary Table S1).

### 3.4. Association with Adverse Histology

Modelled continuously, all six indices were associated with adverse histology in univariable logistic regression (Table 3). Each 1-SD increase in SIRI was associated with more than two-fold higher odds of adverse histology (OR: 2.35, 95% CI: 1.41–3.90), with PIV and SII showing comparable effects; all six univariable associations remained significant after false-discovery-rate correction. After adjustment for age, sex, smoking, multifocality, tumour size and prior recurrence, the SIRI association was attenuated and no longer statistically significant (adjusted OR: 1.69 per 1-SD, 95% CI: 0.92–3.11; *p* = 0.089), while tumour size remained independently associated (OR: 1.05 per mm; *p* = 0.042). In secondary, exploratory analyses of the individual components, SIRI was associated univariably with each component—high grade (OR: 2.00, 95% CI: 1.34–2.98), stage T1 (OR: 1.77, 1.23–2.54) and concomitant CIS (OR: 1.53, 1.08–2.17)—but after the same adjustment none of these associations remained statistically significant (adjusted OR: 1.55, 1.51 and 1.51; *p* = 0.071, 0.068 and 0.069, respectively; Supplementary Table S2).

### 3.5. Recurrence-Free Survival

In the descriptive Kaplan–Meier analysis, values above the Youden cut-offs for SIRI, SII and PIV were associated with worse recurrence-free survival (Supplementary Figures S1 and S2). On univariable Cox regression, each 1-SD increase in SIRI was associated with a 41% higher recurrence hazard (HR: 1.41, 95% CI: 1.19–1.67), comparable to the other indices (HR: 1.34–1.43 per 1-SD for NLR, PLR, SII and PIV; HR: 0.72 for LMR) (Table 4). All six univariable associations remained significant after false-discovery-rate correction. After adjustment for the coarse EAU risk group alone, SIRI remained associated with recurrence (HR: 1.30 per 1-SD, 95% CI: 1.08–1.56; *p* = 0.005). However, in a full clinical risk model (EAU risk group, adjuvant therapy, tumour size, multifocality, prior recurrence and age), SIRI was no longer independently associated with recurrence (adjusted HR: 1.10, 95% CI: 0.86–1.40; *p* = 0.45), and its addition did not improve discrimination: the C-index rose only from 0.663 (95% CI: 0.610–0.719) to 0.664 (0.610–0.720), with an ΔC of 0.001 (95% CI: −0.006 to 0.016; likelihood-ratio test for adding SIRI, *p* = 0.46) and a time-dependent 12-month AUC of 0.709 versus 0.709 (ΔAUC: 0.000, bootstrap 95% CI: −0.009 to 0.022). The dominant predictors in this model were the EAU risk group (HR: 1.91, 95% CI: 1.12–3.26) and tumour size (HR: 1.05 per mm, 95% CI: 1.02–1.08). The variance inflation factor was below 2.7 for all covariates (SIRI: 1.59), a restricted-cubic-spline/quadratic term showed no departure from linearity (*p* = 0.42), the optimism-corrected C-index was 0.631 and the optimism-corrected calibration slope was 0.79 (95% CI: 0.57–1.07). Model coefficients and discrimination are shown in Table 4 and Table 5.

**Figure 3:**
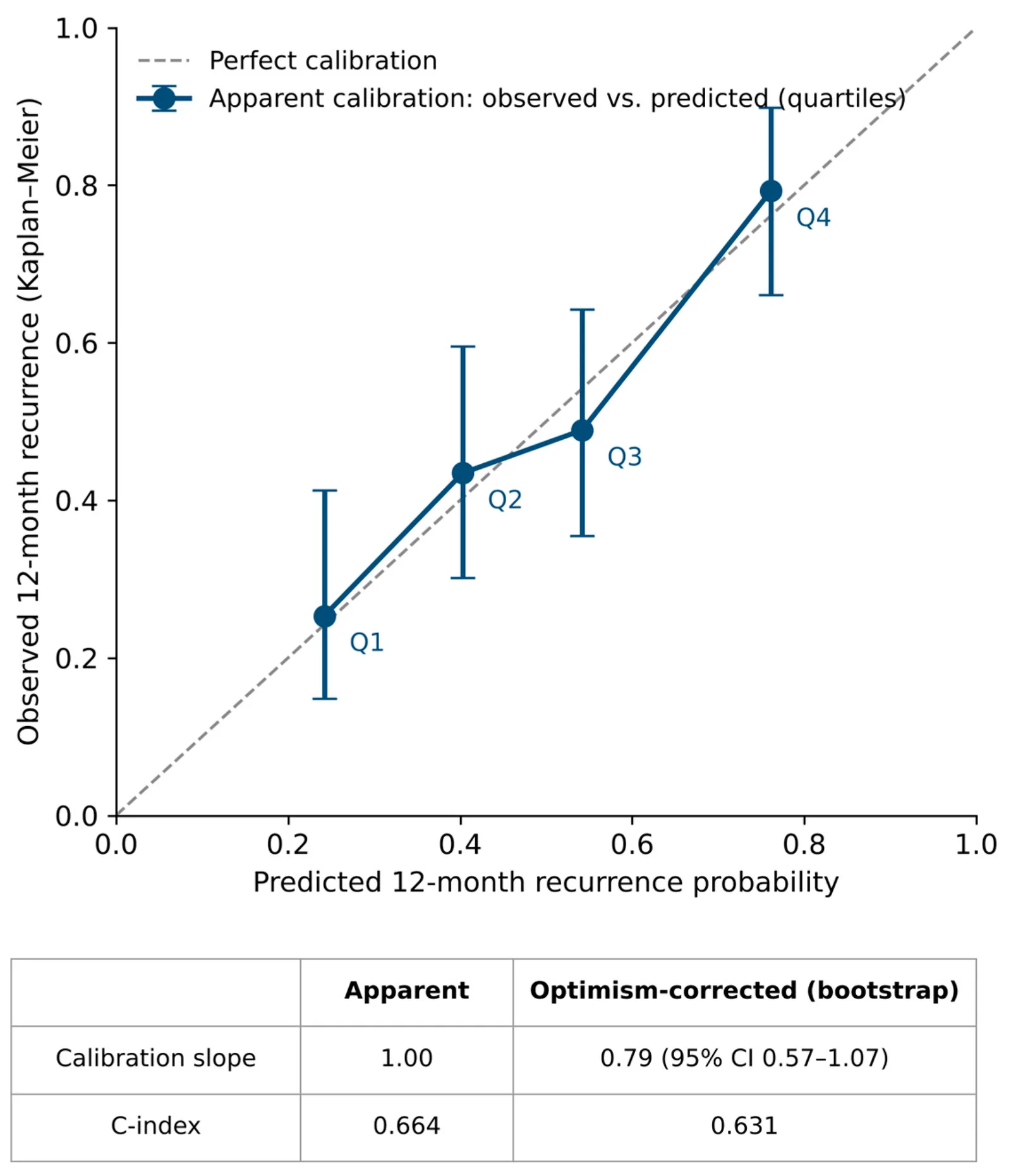
Calibration of the full clinical model, including SIRI, for 12-month recurrence. Observed Kaplan–Meier recurrence (with 95% confidence intervals) is plotted against mean predicted probability across quartiles (Q1–Q4) of predicted risk; the dashed line indicates perfect calibration. The plotted points show apparent calibration (without optimism correction); the table below the plot reports the apparent and bootstrap optimism-corrected calibration slope and C-index.

In sensitivity analyses (Supplementary Table S3), SIRI was not independently associated with recurrence after inverse-probability-of-treatment weighting for adjuvant BCG (HR: 0.92, 95% CI: 0.57–1.48; *p* = 0.73), when the EAU risk group was entered as a binary variable (HR: 1.10, 0.86–1.40; *p* = 0.45), and within adjuvant-treated (HR: 0.99, 0.74–1.31), BCG-treated (HR: 0.99, 0.71–1.39) and high-risk patients (HR: 1.04, 0.75–1.43). Omitting the EAU risk group, so that only three of the variables underlying it (tumour size, multifocality and prior recurrence) remained in the model, gave an HR of 1.13 (0.90–1.44; *p* = 0.30), whereas adding tumour size alone to the EAU risk group reduced the estimate from an HR of 1.30 to 1.14 (0.91–1.44); in 1000 bootstrap resamples of the full model, the median SIRI hazard ratio was 1.10 (2.5th–97.5th percentile: 0.80–1.44) and *p* < 0.05 was reached in 16%. In a granular clinicopathological model, which discriminated better than the EAU risk-group model (C-index: 0.720 vs. 0.663), high grade (HR: 2.88, 1.73–4.78), stage T1 (HR: 2.05, 1.23–3.39) and tumour size (HR: 1.04 per mm, 1.01–1.07) were the dominant predictors, whereas concomitant CIS (HR: 0.98, 0.52–1.87) and SIRI (HR: 1.03, 0.80–1.31; *p* = 0.84) were not; adding SIRI did not change discrimination (ΔC: 0.000, −0.004 to 0.012; Supplementary Tables S3 and S4). Results were also unchanged with log-transformed SIRI (HR: 1.05, 0.77–1.43); with the number of tumours in place of multifocality (HR: 1.09, 0.86–1.38); after additional adjustment for diabetes, body mass index and smoking (HR: 1.10, 0.86–1.41); and with the EAU risk group entered as two indicator variables (HR: 1.10, 0.86–1.40). None of the other five indices was independently associated with recurrence when substituted for SIRI in the full model (HR per 1-SD: NLR: 1.16, 0.92–1.45; PLR: 1.12, 0.89–1.39; LMR: 1.09, 0.80–1.49; SII: 1.12, 0.90–1.40; PIV: 1.07, 0.85–1.35). In logistic regression for recurrence by 12 months (n = 161; 87 events), SIRI was associated with recurrence univariably (OR: 1.75, 1.16–2.63) but not after adjustment (OR: 1.04, 0.66–1.62; *p* = 0.87), and the 12-month AUC was unchanged (0.708 vs. 0.708; DeLong *p* = 0.88). Truncating follow-up at 12 months left the estimate unchanged (HR: 1.10, 0.87–1.41; *p* = 0.42). In apparent calibration, observed 12-month recurrence closely matched predicted risk across quartiles (predicted: 0.24, 0.40, 0.54 and 0.76 vs. observed: 0.25, 0.43, 0.49 and 0.79; Figure 3), although the optimism-corrected calibration slope (0.79) indicated modest overfitting. With 88 events, the study had approximately 71% power to detect a hazard ratio of 1.4 per 1-SD of SIRI and 80% power to detect a hazard ratio of 1.46.

**Table 4:** Cox regression for 12-month recurrence (indices modelled continuously, per 1-SD).

Model/Predictor	HR (95% CI) per 1-SD	*p*
**Univariable**		
NLR	1.43 (1.21–1.70)	<0.001
PLR	1.35 (1.15–1.59)	<0.001
LMR	0.72 (0.54–0.96)	0.024
SII	1.36 (1.18–1.58)	<0.001
SIRI	1.41 (1.19–1.67)	<0.001
PIV	1.34 (1.16–1.55)	<0.001
Full clinical model + SIRI *		
EAU risk group (per level)	1.91 (1.12–3.26)	0.018
Adjuvant chemotherapy (vs. none)	1.27 (0.71–2.29)	0.421
Adjuvant BCG (vs. none)	0.72 (0.34–1.52)	0.396
Tumour size (per mm)	1.05 (1.02–1.08)	0.002
Multifocality	1.33 (0.86–2.05)	0.202
Prior recurrence	1.02 (0.65–1.58)	0.937
Age (per year)	0.986 (0.963–1.009)	0.239
SIRI (per 1-SD)	1.10 (0.86–1.40)	0.451

* Full clinical model. For comparison, SIRI adjusted for the coarse EAU risk group alone gave an HR of 1.30 (1.08–1.56), *p* = 0.005; this did not persist in the full model. The proportional-hazards assumption held for SIRI (Schoenfeld *p* = 0.34) and for all covariates except age (*p* = 0.02); a model stratified by age left the SIRI estimate essentially unchanged (HR: 1.09). The variance inflation factor was below 2.7 for all covariates (SIRI: 1.59). HR, hazard ratio; CI, confidence interval; SD, standard deviation.

**Table 5:** Discrimination and incremental value of SIRI added to the clinical risk model.

Model	C-Index (95% CI)	ΔC vs. Base (95% CI)	Time-Dependent 12 mo AUC
Clinical risk model (base)	0.663 (0.610–0.719)	—	0.709
Clinical + SIRI	0.664 (0.610–0.720)	0.001 (−0.006 to 0.016)	0.709
SIRI alone	0.603 (0.536–0.668)	—	0.650

Bootstrap 2000 resamples (percentile intervals; for ΔC, both models were refitted in each resample); likelihood-ratio test for adding SIRI χ^2^ = 0.54 (1 df), *p* = 0.46; ΔC bootstrap *p* = 0.70. Time-dependent 12-month AUC estimated with inverse-probability-of-censoring weighting, with patients event-free at 12 months as controls; ΔAUC 0.000 (bootstrap 95% CI: −0.009 to 0.022, refitted models). The optimism-corrected C-index of the base + SIRI model was 0.631, with an optimism-corrected calibration slope of 0.79 (95% CI: 0.57–1.07). Base model: EAU risk group, adjuvant therapy, tumour size, multifocality, prior recurrence, age. AUC, area under the curve.

## 4. Discussion

In this single-centre cohort of 180 NMIBC patients treated by TURBT under a uniform immediate-instillation protocol, all six preoperative inflammatory indices showed univariable associations with adverse histology and 12-month recurrence when modelled as continuous variables. SIRI showed a univariable signal comparable to that of the other indices, but it was not independently associated with recurrence after adjustment for a full clinical risk model, and it did not improve discrimination beyond established clinical variables. Although these indices are inexpensive and derived from a routine complete blood count, this advantage matters only if they add prognostic information, which SIRI did not demonstrably do here.

The contribution of this study is primarily corrective rather than the identification of a new marker. Most published series in NMIBC and bladder cancer have reported positive associations [16,17,21,22,23], one of them after propensity-score matching [22], frequently using thresholds derived and tested with the same data, and a recent meta-analysis across urological malignancies reached similar conclusions [24]; data-derived thresholds are prone to optimism and, where adjustment is limited, to residual confounding by tumour characteristics. By modelling the indices continuously, quantifying incremental discrimination with bootstrap confidence intervals and testing the results across complementary sensitivity analyses, we show that a consistent univariable signal does not translate into independent prognostic information. Such negative findings are under-reported yet necessary, because inexpensive blood-based markers are readily incorporated into risk tools before their incremental value has been established.

Our findings should be read against a growing literature. Recent studies in NMIBC [16,17,22,23,25] and in bladder or urological cancer more broadly [21,24] have reported associations between SIRI or SII and recurrence, progression, or treatment response, but findings vary according to population, endpoint, treatment exposure and statistical approach. In particular, recent work in NMIBC has used different thresholds and has not consistently evaluated the incremental value of inflammatory indices beyond established clinical risk models. PIV, the most composite index, performed comparably to SIRI in our analysis, consistent with pilot NMIBC data [25]. These findings also complement our earlier work on determinants of TURBT quality: whereas control of the obturator reflex by obturator nerve block was associated with better specimen quality and lower early recurrence in lateral-wall tumours [6], a preoperative systemic inflammatory index did not add independent prognostic information once established clinical variables were considered. The present study therefore contributes a continuous-variable analysis focused on a uniformly staged NMIBC cohort, with explicit assessment of whether SIRI adds prognostic information beyond conventional risk stratification.

A methodologically important observation is the divergent behaviour of SIRI across multivariable models. This pattern is biologically coherent: the systemic inflammatory response is partly a downstream reflection of tumour aggressiveness, so inflammatory indices and adverse histology carry overlapping prognostic information. A clinically relevant question is whether a preoperatively available marker refines the estimate provided by the standard tool. When SIRI was added only to the coarse three-level EAU risk group it appeared to retain independent significance (HR: 1.30 per 1-SD); however, this apparent added value did not survive adjustment for a full clinical risk model, in which SIRI was no longer independently associated with recurrence (HR: 1.10, *p* = 0.45) and produced no meaningful gain in discrimination (ΔC: 0.001, 95% CI: −0.006 to 0.016). The earlier signal therefore largely reflected residual confounding by tumour characteristics not captured by the three-level category, principally tumour size, the addition of which alone reduced the SIRI estimate to an HR of 1.14, rather than independent prognostic information carried by SIRI. For the histological components, adjustment attenuated the associations of SIRI with high grade and stage T1 (OR: 2.00 to 1.55 and 1.77 to 1.51), whereas that with CIS was essentially unchanged but imprecise (1.53 to 1.51); none remained statistically significant. SIRI also remained non-significant after propensity weighting for adjuvant treatment and in homogeneously treated subgroups. The conclusion that SIRI adds no demonstrable independent prognostic value for recurrence rests on three concordant observations: the adjusted hazard ratio was close to 1, although the upper limit of its confidence interval (1.40) approached the univariable estimate (1.41); the upper confidence limit for the change in C-index (0.016) remained below 0.02, a gain unlikely to be clinically meaningful; and the adjusted hazard and odds ratios remained between 0.92 and 1.14 across all sensitivity analyses (Supplementary Table S3), with no gain in discrimination where this was assessed (granular model, ΔC: 0.000; logistic model, AUC: 0.708 vs. 0.708). For adverse histology, the adjusted estimate (OR: 1.69, 0.92–3.11) was imprecise, so these data show only that an independent association was not demonstrated.

The descriptive cut-offs (Supplementary Table S1 and Figures S1 and S2) should be interpreted cautiously. They were derived internally using the Youden index and therefore may be optimistically biased and are not clinically validated thresholds. Their heterogeneity across studies likely reflects differences in populations, analyser platforms, endpoints and statistical methods. This variability argues for continuous modelling and external validation rather than transfer of a single cut-off between centres.

Strengths of this study include a uniform surgical protocol, adjustment for adjuvant intravesical therapy, complete preoperative blood counts, a prespecified composite adverse-histology endpoint, continuous modelling that avoids data-driven dichotomisation, and an explicit assessment of incremental discrimination beyond clinical risk stratification. Limitations should be acknowledged. The retrospective, single-centre design limits generalisability and leaves residual confounding possible. A single preoperative blood sample cannot capture the dynamic nature of systemic inflammation. Intercurrent infection, physiological fluctuation or subclinical inflammatory states may transiently alter leucocyte and platelet counts, and serial perioperative measurements would be required to characterise this variability; random within-person variation of this kind would be expected to attenuate associations towards the null (regression dilution), which is relevant to the interpretation of a null finding. Follow-up was limited to approximately 12 months (maximum: 13 months), so progression and longer-term recurrence were not assessed. Our conclusions are therefore restricted to 12-month recurrence, and progression and late recurrence require longer prospective follow-up. Histopathology was assessed locally without independent central review; misclassification of grade or stage cannot be excluded and may have affected both the adverse-histology endpoint and EAU risk-group assignment, representing a major potential source of bias. Immediate intravesical instillation was administered to all patients irrespective of risk, reflecting institutional practice rather than a fully risk-adapted contemporary approach and potentially limiting generalisability. Treatment allocation occurred after TURBT and was influenced by clinical and pathological characteristics; adjustment for adjuvant therapy should therefore be interpreted as an associational adjustment rather than a causal estimate. Propensity weighting improved but did not fully achieve balance in the EAU risk group (standardised mean difference: 0.29 after weighting), because BCG was given predominantly to high-risk patients (52 out of 66) and never to low-risk patients, and every high-risk patient received an adjuvant course (Supplementary Table S5), so the positivity assumption was violated in the low-risk stratum; the IPTW analysis is therefore supportive rather than a definitive control of confounding by indication. The optimism-corrected calibration slope (0.79) indicates modest overfitting, so predicted risks would require recalibration before use in other settings. Internally derived ROC thresholds may be optimistic, and the incremental discrimination beyond the clinical risk model was negligible, with a confidence interval including no improvement. Exclusion at eligibility of patients with incomplete blood counts or follow-up (n = 6) may have introduced minor selection bias. Medication use was abstracted retrospectively from the medical records; undocumented use of anti-inflammatory, antiplatelet or lipid-lowering drugs (for example, over-the-counter analgesics) cannot be excluded and could have altered leucocyte counts or the systemic inflammatory state. If unrelated to tumour biology, such misclassification would be expected to attenuate rather than create associations, but residual confounding by medication remains possible. Adjuvant intravesical therapy was individualised and partly constrained by drug availability rather than protocolised, and a subset of intermediate-risk patients did not receive a guideline-recommended adjuvant course, so residual confounding by treatment cannot be excluded despite adjustment. In sensitivity analyses restricted to homogeneously treated subgroups, SIRI remained non-significant (adjuvant-treated patients, adjusted HR: 0.99; BCG-treated, HR: 0.99; high-risk patients, HR: 1.04; Supplementary Table S3), so the absence of an independent association was not explained by treatment heterogeneity, although these subgroups (37–67 events) could not exclude a moderate effect. The study was not powered to exclude a moderate independent effect of SIRI (approximately 71% power for a hazard ratio of 1.4 per 1-SD and 80% power for 1.46), and the upper confidence limit of the adjusted hazard ratio (1.40) is close to the univariable estimate; post hoc power is of limited value compared with these confidence intervals. The full model estimated eight parameters (11 events per parameter) and the granular model eleven (8.0 events per parameter). Vital-status data were unavailable, so competing mortality could not be modelled with competing-risk methods, and the magnitude and direction of any resulting bias cannot be quantified; future studies should collect vital-status data. Finally, estimates derived in a single cohort require prospective, multicentre external validation, which is the principal direction for future work.

## 5. Conclusions

Preoperative systemic inflammatory indices, including SIRI, were associated univariably with adverse histology and 12-month recurrence in this NMIBC cohort. However, SIRI was not independently associated with either endpoint after adjustment for established clinical variables and added negligible discrimination for recurrence (ΔC: 0.001, 95% CI: −0.006 to 0.016); none of the other indices was independently associated with recurrence when substituted for SIRI. These indices do not replace, and in this cohort did not meaningfully complement, pathological staging or established risk models, and they are not ready for clinical use. Because the clinical model showed modest overfitting (optimism-corrected calibration slope: 0.79), neither these indices nor the model should be applied clinically without prospective, multicentre external validation and recalibration.

## Figures and Tables

**Table 1:** Baseline characteristics of the cohort, overall and by recurrence status during follow-up.

Characteristic	Overall (n = 180)	Recurrence (n = 88)	No Recurrence (n = 92)
Age, years—median (IQR)	68 (62–74)	66 (60–73)	71 (63–76)
Male sex, n (%)	136 (75.6)	62 (70)	74 (80)
Current smoker, n (%)	80 (44.4)	35 (40)	45 (49)
Diabetes, n (%)	34 (18.9)	18 (20)	16 (17)
Tumour size, mm—median (IQR)	14 (8–23)	18.5 (11–25)	12 (7–18)
Multifocal, n (%)	76 (42.2)	44 (50)	32 (35)
High grade, n (%)	84 (46.7)	58 (66)	26 (28)
Stage T1, n (%)	60 (33.3)	40 (45)	20 (22)
Concomitant CIS, n (%)	33 (18.3)	15 (17)	18 (20)
Recurrent tumour, n (%)	60 (33.3)	31 (35)	29 (32)
EAU risk group—low, n (%)	20 (11.1)	4 (5)	16 (17)
EAU risk group—intermediate, n (%)	100 (55.6)	46 (52)	54 (59)
EAU risk group—high, n (%)	60 (33.3)	38 (43)	22 (24)
Adjuvant BCG, n (%)	66 (36.7)	37 (42)	29 (32)
Adjuvant chemotherapy, n (%)	63 (35.0)	30 (34)	33 (36)
No further adjuvant therapy, n (%)	51 (28.3)	21 (24)	30 (33)

IQR, interquartile range; CIS, carcinoma in situ; EAU, European Association of Urology. *p*-values: age 0.038, tumour size < 0.001, multifocality 0.055, high grade < 0.001, stage T1 0.001, EAU risk group 0.002, adjuvant therapy 0.271; remaining comparisons not significant.

**Table 2:** Preoperative inflammatory indices by recurrence status during follow-up (Mann–Whitney U test).

Index	Recurrence, Median (IQR)	No Recurrence, Median (IQR)	*p*
NLR	2.24 (1.67–3.23)	1.83 (1.32–2.40)	<0.001
PLR	128.9 (96.8–181.2)	103.7 (86.5–142.9)	0.007
LMR	3.35 (2.34–4.92)	4.03 (3.15–6.09)	0.002
SII	559 (395–848)	416 (299–652)	<0.001
SIRI	1.37 (0.84–2.22)	0.99 (0.56–1.47)	<0.001
PIV	341.5 (171.6–620.6)	216.3 (121.6–382.5)	<0.001

Abbreviations as defined in the text.

**Table 3:** Logistic regression for adverse histology (indices modelled continuously, per 1-SD).

Predictor	OR (95% CI) per 1-SD	*p*
**Univariable**		
NLR	2.20 (1.36–3.57)	0.001
PLR	1.96 (1.20–3.19)	0.007
LMR	0.58 (0.42–0.81)	0.001
SII	2.53 (1.40–4.56)	0.002
SIRI	2.35 (1.41–3.90)	0.001
PIV	2.75 (1.47–5.14)	0.002
Multivariable (SIRI + clinical) *		
SIRI	1.69 (0.92–3.11)	0.089
Tumour size (per mm)	1.055 (1.002–1.110)	0.042
Prior recurrence	0.46 (0.23–0.92)	0.029

* Adjusted for age, sex, smoking, multifocality, tumour size and prior recurrence. OR, odds ratio; CI, confidence interval; SD, standard deviation.

## Data Availability

The data presented in this study are available from the corresponding author on reasonable request; they are not publicly available due to privacy restrictions.

## Supplementary Materials

The following supporting information can be downloaded at https://www.mdpi.com/article/10.3390/jcm15218536/s1, Checklist S1: STROBE Statement—checklist of items that should be included in reports of cohort studies; Table S1: ROC discrimination and Youden-derived descriptive cut-offs for both endpoints; Figure S1: Kaplan–Meier recurrence-free survival stratified by preoperative SIRI (descriptive cut-off 1.63; log-rank *p* < 0.001), with numbers at risk; Figure S2: Kaplan–Meier recurrence-free survival by (A) SII and (B) PIV (descriptive cut-offs 535 and 323; log-rank *p* = 0.001 and *p* < 0.001, respectively), with numbers at risk; Table S2: Association of SIRI (per 1-SD) with the individual components of the composite adverse-histology endpoint (logistic regression); Table S3: Sensitivity analyses for 12-month recurrence: SIRI (per 1-SD) under alternative specifications, and the other indices substituted for SIRI (Cox models unless stated otherwise); Table S4: Granular multivariable Cox model for 12-month recurrence (n = 180; 88 events; 11 parameters, 8.0 events per parameter); Table S5: Adjuvant intravesical therapy by EAU risk group.
