# Supplementary material for: Preoperative Systemic Inflammatory Indices (SII, SIRI and PIV) Associated with Adverse Histology and 12-Month Recurrence After Transurethral Resection of Non-Muscle-Invasive Bladder Cancer
Source: J Clin Med. 2026 Nov 2;15(21):8536. doi: 10.3390/jcm15218536 (PMC13763628; doi:10.3390/jcm15218536)
Supplement: Supplementary file 1 [file jcm-15-08536-s001.zip › jcm-4611565-supplementary.pdf]

Supplementary Material

Preoperative Systemic Inflammatory Indices (SII, SIRI and PIV) Associated with Adverse Histology and 12-Month Recurrence after Transurethral Resection of Non-Muscle-Invasive Bladder Cancer

Contents: Checklist S1; Table S1; Figure S1; Figure S2; Table S2; Table S3; Table S4; Table S5.

Checklist S1. STROBE Statement—checklist of items that should be included in reports of cohort studies.

Section	Item	Recommendation	Location in manuscript
Title and abstract	1a	Indicate the study's design with a commonly used term in the title or the abstract	Title; Abstract ("We retrospectively analysed…")
	1b	Provide in the abstract an informative and balanced summary of what was done and what was found	Abstract (structured: Background/Objectives, Methods, Results, Conclusions)
Introduction			
Background/rationale	2	Explain the scientific background and rationale for the investigation being reported	Introduction (paragraphs 1–3)
Objectives	3	State specific objectives, including any prespecified hypotheses	Introduction (final paragraph)
Methods			
Study design	4	Present key elements of study design early in the paper	Section 2.1
Setting	5	Describe the setting, locations, and relevant dates, including periods of recruitment, exposure, follow-up, and data collection	Sections 2.1 and 2.2
Participants	6a	Give the eligibility criteria, and the sources and methods of selection of participants; describe methods of follow-up	Section 2.1 (eligibility and exclusion criteria); Section 3.1 and Figure 1 (study flow)
Variables	7	Clearly define all outcomes, exposures, predictors, potential confounders, and effect modifiers	Sections 2.3 (inflammatory indices) and 2.4 (endpoints)
Data sources/measurement	8	For each variable of interest, give sources of data and details of methods of assessment	Section 2.3
Bias	9	Describe any efforts to address potential sources of bias	Section 2.1 (exclusion of conditions affecting blood counts); Section 2.5 (adjustment and sensitivity analyses); Discussion (limitations)
Study size	10	Explain how the study size was arrived at	Section 2.5 and Discussion (post hoc power: approximately 71% for HR 1.4 and 80% for HR 1.46 per 1-SD)
Quantitative variables	11	Explain how quantitative variables were handled in the analyses; describe groupings and why	Section 2.5 (continuous per 1-SD modelling; descriptive Youden cut-offs)
Statistical methods	12	Describe all statistical methods, confounding, subgroups, missing data, and sensitivity analyses	Section 2.5 (models, missing data, censoring and sensitivity analyses)
Results			
Participants	13	Report numbers of individuals at each stage; reasons for non-participation	Section 3.1; Figure 1
Descriptive data	14	Give characteristics of study participants and information on exposures and potential confounders	Section 3.1; Table 1
Outcome data	15	Report numbers of outcome events or summary measures over time	Sections 3.1–3.5; Tables 2–5; Figures 1–3

Main results	16	Give unadjusted and confounder-adjusted estimates and their precision (e.g., 95% CI)	Sections 3.4 and 3.5; Tables 3–5 (unadjusted and adjusted OR/HR with 95% CI)
Other analyses	17	Report other analyses done (subgroups, interactions, sensitivity, incremental value)	Section 3.3 (ROC analysis); Section 3.5 (incremental C-index, sensitivity analyses); Supplementary Tables S2–S5
Discussion			
Key results	18	Summarise key results with reference to study objectives	Discussion (first paragraph)
Limitations	19	Discuss limitations, taking into account sources of potential bias or imprecision	Discussion (limitations paragraph)
Interpretation	20	Give a cautious overall interpretation of results considering objectives, limitations, and other evidence	Discussion; Conclusions
Generalisability	21	Discuss the generalisability (external validity) of the study results	Discussion (single-centre design, uniform immediate instillation, need for external validation)
Other information			
Funding	22	Give the source of funding and the role of the funders	Funding statement

Adapted from von Elm E, et al. The Strengthening the Reporting of Observational Studies in Epidemiology (STROBE) statement. BMJ 2007;335:806–808. Section numbers refer to the published article.

Table S1. ROC discrimination and Youden-derived descriptive cut-offs for both endpoints.

Index	Adverse histology AUC	Adverse histology cut-off	12-month recurrence AUC	12-month recurrence cut-off	Sensitivity / specificity †
NLR	0.67	> 1.85	0.64	> 1.97	0.64 / 0.59
PLR	0.62	> 121	0.62	> 116	0.64 / 0.61
LMR	0.65	≤ 3.09	0.63	≤ 3.46	0.56 / 0.68
SII	0.67	> 547	0.65	> 535	0.58 / 0.68
SIRI	0.67	> 1.13	0.65	> 1.63	0.43 / 0.83
PIV	0.68	> 314	0.64	> 323	0.58 / 0.70

AUC, area under the receiver operating characteristic curve. † Sensitivity/specificity at the 12-month recurrence cut-off. Cut-offs were derived internally with the Youden index, are descriptive only and potentially optimistic, were not used in any regression model, and have not been externally validated; they should not be transferred between centres. All association testing in the main text used continuous (per 1-SD) indices. Recurrence ROC analyses compared the 88 patients with recurrence with the 92 without (including 19 censored before 12 months without recurrence); restricted to the 161 patients with an ascertainable 12-month status (87 with and 74 without recurrence), AUCs were similar (NLR 0.64, PLR 0.62, LMR 0.64, SII 0.64, SIRI 0.65, PIV 0.65) and the SIRI cut-off was unchanged (> 1.63).

Figure S1. Kaplan–Meier recurrence-free survival stratified by preoperative SIRI (descriptive cut-off 1.63; log-rank p < 0.001), with numbers at risk.

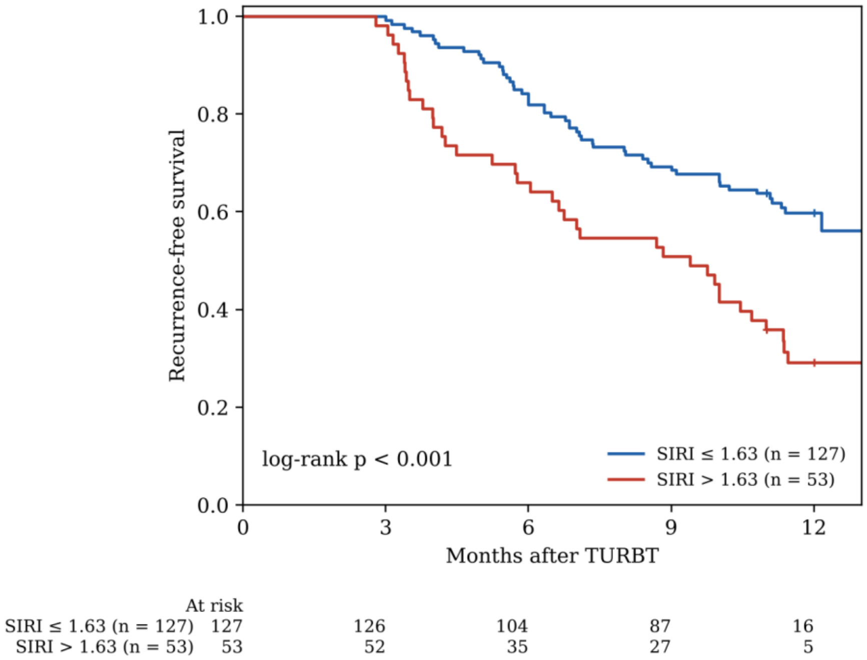

The cut-off is internally derived and descriptive (see Table S1); inference is based on continuous modelling (main text, Table 4). Tick marks indicate censored observations. Log-rank p-values at data-derived cut-offs are optimistic and are shown for description only.

Figure S2. Kaplan–Meier recurrence-free survival by (A) SII and (B) PIV (descriptive cut-offs 535 and 323; log-rank p = 0.001 and p < 0.001, respectively), with numbers at risk.

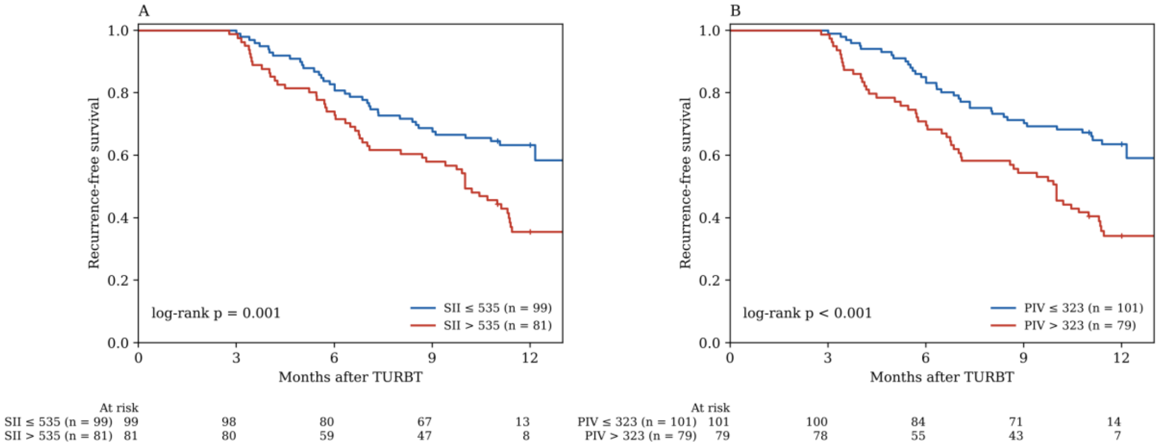

Cut-offs are internally derived and descriptive (see Table S1). Tick marks indicate censored observations. Log-rank p-values at data-derived cut-offs are optimistic and are shown for description only.

Table S2. Association of SIRI (per 1-SD) with the individual components of the composite adverse-histology endpoint (logistic regression).

Component	Events, n	Univariable OR (95% CI)	p	Adjusted OR (95% CI) *	p
High grade	84	2.00 (1.34–2.98)	0.001	1.55 (0.96–2.51)	0.071
Stage T1	60	1.77 (1.23–2.54)	0.002	1.51 (0.97–2.36)	0.068
Concomitant CIS	33	1.53 (1.08–2.17)	0.017	1.51 (0.97–2.37)	0.069

* Adjusted for age, sex, current smoking, multifocality, tumour size and prior recurrence (same covariates as Table 3 of the main text). n = 180. CIS, carcinoma in situ; OR, odds ratio; CI, confidence interval; SD, standard deviation.

Table S3. Sensitivity analyses for 12-month recurrence: SIRI (per 1-SD) under alternative specifications, and the other indices substituted for SIRI (Cox models unless stated otherwise).

Analysis	Patients (events)	HR (95% CI) per 1-SD	p
Primary full clinical model *	180 (88)	1.10 (0.86–1.40)	0.45
IPTW for adjuvant BCG †	180 (88)	0.92 (0.57–1.48)	0.73
EAU risk group entered as binary (high vs. low/intermediate) ‡	180 (88)	1.10 (0.86–1.40)	0.45
Adjuvant-treated patients (BCG or chemotherapy) §	129 (67)	0.99 (0.74–1.31)	0.93
BCG-treated patients §	66 (37)	0.99 (0.71–1.39)	0.96
High-risk (EAU) patients §	60 (38)	1.04 (0.75–1.43)	0.83
Granular model: individual histological and clinical variables instead of EAU risk group ‖	180 (88)	1.03 (0.80–1.31)	0.84
SIRI log-transformed (per 1-SD of log SIRI) ‡	180 (88)	1.05 (0.77–1.43)	0.74
Number of tumours (count) instead of multifocality ‡	180 (88)	1.09 (0.86–1.38)	0.48
Additionally adjusted for diabetes, BMI and smoking ‡	180 (88)	1.10 (0.86–1.41)	0.46
Logistic regression, binary recurrence by 12 months	161 (87)	OR 1.04 (0.66–1.62)	0.87
Follow-up truncated at 12 months (one later recurrence censored) ‡	180 (87)	1.10 (0.87–1.41)	0.42
EAU risk group omitted (underlying variables only) **	180 (88)	1.13 (0.90–1.44)	0.30
EAU risk group and tumour size only (with SIRI) ††	180 (88)	1.14 (0.91–1.44)	0.25
EAU risk group entered as two indicator variables ‡	180 (88)	1.10 (0.86–1.40)	0.45
Other index in place of SIRI in the primary model ‡‡: NLR	180 (88)	1.16 (0.92–1.45)	0.21

PLR	180 (88)	1.12 (0.89–1.39)	0.34
LMR	180 (88)	1.09 (0.80–1.49)	0.59
SII	180 (88)	1.12 (0.90–1.40)	0.29
PIV	180 (88)	1.07 (0.85–1.35)	0.55

* EAU risk group (ordinal), adjuvant therapy (none/chemotherapy/BCG), tumour size, multifocality, prior recurrence and age. † Inverse-probability-of-treatment weighting; propensity for adjuvant BCG estimated by logistic regression on EAU risk group, tumour size, multifocality, prior recurrence, age and SIRI; weights truncated at the 99th percentile; robust (sandwich) variance. After weighting, standardised mean differences were ≤ 0.12 for all covariates except the EAU risk group (1.99 before, 0.29 after weighting), reflecting that BCG was given predominantly to high-risk patients (52 of 66) and never to low-risk patients (positivity violated in the low-risk stratum; Table S5); the weighted outcome model therefore also adjusted for EAU risk group and the other clinical covariates. ‡ Otherwise identical to the primary model. § Unpenalised Cox models including the primary-model covariates that varied within the subgroup. ‖ Age, sex, tumour size, number of tumours, prior recurrence, grade, stage, concomitant CIS and adjuvant therapy; C-index 0.720 with and without SIRI (ΔC 0.000, bootstrap 95% CI −0.004 to 0.012); full coefficients in Table S4. ¶ Patients with ≥ 12 months of follow-up or recurrence within 12 months (19 patients with shorter event-free follow-up excluded); same covariates as the primary model; univariable OR 1.75 (1.16–2.63); AUC 0.708 with and without SIRI (DeLong p = 0.88). ** Adjuvant therapy, tumour size, multifocality, prior recurrence and age, without the EAU risk group, to assess redundancy between the risk group and the variables underlying it; in 1000 bootstrap resamples of the primary model, SIRI reached p < 0.05 in 16% (median HR 1.10, 2.5th–97.5th percentile 0.80–1.44). †† Primary-model covariates reduced to the EAU risk group and tumour size, to show that adding tumour size alone accounts for most of the attenuation from the EAU-risk-group-only estimate (HR 1.30). ‡‡ Each index per 1-SD, replacing SIRI in the primary model; HR shown is for the substituted index. HR, hazard ratio; OR, odds ratio; CI, confidence interval; IPTW, inverse-probability-of-treatment weighting; BMI, body mass index; EAU, European Association of Urology.

Table S4. Granular multivariable Cox model for 12-month recurrence (n = 180; 88 events; 11 parameters, 8.0 events per parameter).

Variable	HR (95% CI)	p
Age (per year)	0.99 (0.97–1.02)	0.44
Male sex	0.73 (0.46–1.17)	0.19
Tumour size (per mm)	1.04 (1.01–1.07)	0.006
Number of tumours (per tumour)	1.10 (0.97–1.24)	0.13
Recurrent (vs. primary) tumour	1.11 (0.70–1.77)	0.66
High grade (vs. low grade)	2.88 (1.73–4.78)	< 0.001
Stage T1 (vs. Ta)	2.05 (1.23–3.39)	0.006
Concomitant CIS	0.98 (0.52–1.87)	0.96
Adjuvant chemotherapy (vs. none)	1.27 (0.71–2.29)	0.42
Adjuvant BCG (vs. none)	0.70 (0.35–1.39)	0.31
SIRI (per 1-SD)	1.03 (0.80–1.31)	0.84

Schoenfeld residual tests showed no departure from proportional hazards for SIRI (p = 0.23) or other covariates except age (p = 0.03); stratifying the model by age tertiles left the SIRI estimate unchanged (HR 1.02, 0.79–1.32; p = 0.89). C-index 0.720 (0.720 without SIRI; clinical-only model with EAU risk group, 0.663). BCG, bacillus Calmette–Guérin; CI, confidence interval; CIS, carcinoma in situ; HR, hazard ratio; SD, standard deviation.

Table S5. Adjuvant intravesical therapy by EAU risk group.

EAU risk group	BCG, n (%)	Chemotherapy, n (%)	None, n (%)	Total
Low	0 (0)	8 (40.0)	12 (60.0)	20
Intermediate	14 (14.0)	47 (47.0)	39 (39.0)	100
High	52 (86.7)	8 (13.3)	0 (0)	60
Total	66 (36.7)	63 (35.0)	51 (28.3)	180

All patients additionally received a single immediate postoperative instillation. Allocation of adjuvant therapy followed baseline risk (confounding by indication): BCG was given predominantly to high-risk patients (52 of 66) and never to low-risk patients, whereas every high-risk patient received an adjuvant course. Inverse-probability-of-treatment weighting reduced the standardised mean difference for the EAU risk group from 1.99 to 0.29, so residual imbalance remained; the IPTW analysis in Table S3 is therefore supportive sensitivity evidence rather than a definitive control of confounding. BCG, bacillus Calmette–Guérin; EAU, European Association of Urology.
